# Segue: Brief summaries of articles on pertinent emerging issues published elsewhere.

**DOI:** 10.3201/eid1005.040202

**Published:** 2004-05

**Authors:** Joseph E. McDade

**Affiliations:** Section coordinator

## Population Genetics

### Population Genetics of *Cryptosporidium parvum*

Research on the population genetics of *Cryptosporidium parvum* is showing exciting developments. A study of numerous *C. parvum* isolates of human and animal origin collected in a small area of Scotland has uncovered differences in the epidemiology of what was originally referred to as *C. parvum* type 1 (also, anthroponotic type) and type 2 (zoonotic type), a designation recently changed to *C. hominis* and *C. parvum.* This study, based on fingerprints obtained from eight polymorphic genetic markers, showed striking differences between the population structure of these species; a clonal *C. hominis* population and a more complex *C. parvum* population. The population structure of *C. hominis* was consistent with the epidemic nature of human infections where a small number of genotypes predominate. In contrast, *C. parvum* (type 2) genetic fingerprints showed evidence of random mating among genetically diverse parasites. Evidence for partitioning of the species according to host (human and bovine) was also inferred from these data. Two questions we are left with are whether these observations are specific to this particular location, where human transmission is relatively infrequent, and whether regions with high prevalence of human cryptosporidiosis will show more complex structures in both species. If substructuring into human and bovine derived *C. parvum* (type 2) is consistently observed, the potential for zoonotic transmission of *C. parvum* may also have to be reexamined (*1*).

## Diagnostic Virology

### Role for Arrays in Clinical Virology: Fact or Fiction?

Polymerase chain reaction (PCR) detection of genomic DNA or RNA has become an indispensable tool for the diagnosis and surveillance of viral disease. Perhaps the biggest drawback of PCR, though, is that only one or a few viruses can be searched for in a single test. DNA chips or microarrays have the potential to overcome this disadvantage and can provide a near-patient test that identifies both known viruses and those causing newly emerging diseases such as SARS. For this potential to be realized, however, the PCR techniques capable of amplifying any adventitious sequence in a clinical specimen and the microarraying hybridization and detection technologies necessary for obtaining rapid and reproducible results need to converge. The arrays that have already been developed for use in virology point the way forward (*2*).

## Molecular Epidemiology

### Multilocus Sequence Typing and the Evolution of Methicillin-resistant *Staphylococcus aureus*

Methicillin-resistant *Staphylococcus aureus* (MRSA) continue to adapt to the selective pressure of antimicrobial agents and to exploit new niches, as evidenced by the recent isolation of strains with high-level vancomycin resistance and the emergence of MRSA as a community pathogen. The combined use of the bacterial genotyping technique, multilocus sequence typing (MLST), and characterization of the mobile methicillin-resistance determinant, staphylococcal chromosomal cassette mec (SCCmec), has provided new insights into MRSA strain nomenclature, evolution, and epidemiology. The first MRSA emerged when SCCmec was acquired by an epidemic methicillin-susceptible strain prevalent in Europe. Acquisition of SCCmec by other successful strains has led to the emergence of at least 11 major epidemic MRSA strains belonging to five distinct lineages with a global geographic distribution. These five lineages have evolved both hospital-acquired and community-acquired MRSA, but some of the newly emerging community strains descend from other lineages (*3*).

## Vaccines

### Mimicking Live Flavivirus Immunization with a Noninfectious RNA Vaccine

A new genetic vaccine against flaviviruses is presented that can stimulate a comprehensive immune response similar to a live vaccine but with the safety profile of an inactivated vaccine. The principle is based on the use of noninfectious but replication-competent genomic RNA. This vaccine mimics live viral infection, although there is no spread of virus in the body. The complete replication complex is expressed, including all viral nonstructural proteins known to be targets of the humoral and cellular immune response. In addition, subviral particles consisting of the viral surface proteins prM/M and E are produced and presented to the immune system. The proof-of-principle is demonstrated with tick-borne encephalitis virus (TBEV). Because of the close genetic relationship among members of the genus *Flavivirus*, this principle presumably can also be applied to other pathogens of worldwide medical importance, such as dengue viruses, Japanese encephalitis virus, yellow fever virus, and West Nile virus. The vaccine consists of in vitro synthesized genomic RNA, genetically modified to abolish viral infectivity but to provide ample production and release of subviral particles. Gene-gun mediated injection of this experimental TBEV vaccine into adult mice is shown to yield a neutralizing and protective immune response (*4*).

## Ecology

### Bacterial Biofilms: Prokaryotic Adventures in Multicellularity

Three-dimensional bacterial biofilm microstructures display multicellular characteristics in common with higher organisms, such as cell death and differentiation during development, which carry several medical and evolutionary implications. Biofilm microstructures appear to enhance bacterial tolerance to a number of stresses, including antimicrobial agents; determinants of multicellularity, such as cell-cell signaling, are necessary for this tolerance. Processes of microcolony development and differentiation are therefore of particular interest as targets for novel strategies to control biofilms (*5*).

## Pathogenesis

### CCR5 and CXCR4 Co-receptors in HIV Infection

This article examined the central role played by co-receptor expression and usage in the transmission and pathogenic effects of HIV-1 infection of humans. The review contains a discussion of the HIV-1 phenotypic variants defined by their use of the CCR5 or CXCR4 co-receptors. How the different cellular tropism patterns of these viral variants influence how and where HIV-1 replicates in vivo is also discussed, with emphasis on the thymus and gut-associated lymphoid tissues. The review also contains a consideration of the possible outcomes of the use of co-receptor antagonists as drugs to treat HIV-1 infection in vivo (*6*).
